# Was It Poisoning by Chloroform?

**Published:** 1879-10

**Authors:** P. O’Connell

**Affiliations:** Chicago


### Clinical Reports.

ARTICLE VI.

NOTES FROM PRIVATE PRACTICE.

*Was it Poisoning by Chloroform?*

On Thursday evening, August 14, 1879, R. S., aged 27, single, spare habit, and healthy, purchased a fluid ounce of chloroform. For a few weeks previous he had been despondent, and drank rather freely of both alcohol and absinthe. In three letters, pronounced to be in his handwriting, to as many different persons, he expressed a determination to commit suicide. It is not known what use he made of the chloroform. He is supposed to have taken it internally.

Although seemingly well, he remained in bed on Saturday, August 16, and at noon took a cup of tea with a small piece of toast. About two hours afterward an empty ounce vial, labelled chloroform, was found on his bed. He was thought to be in a critical condition. The writer saw him at 3 p. m., but found him fully conscious, though pale, apparently weak and dull. There was no odor of chloroform from the breath. He was lying on the left side; eyes closed, pupils dilated, but readily responsive to light. Pulse 72, full and soft — normal probably — respirations full, deep and quiet; temperature normal; fingers and toes rather cold. He refused to talk either from real or feigned soreness of the throat. Indeed, a strong suspicion was entertained that the soreness of the throat was more pretence than reality in order to avoid being questioned. The few words he did speak were uttered in a very hoarse whisper. Nothing amiss could be seen in the mouth. He indicated with his finger that the soreness began just behind the larynx, and extended down to the epigastrium. Deglutition seemed both painful and difficult. There was a little tenderness at the epigastrium, but no sickness of stomach at any time. There was no danger then ; and none was apprehended.

He admitted, but subsequently denied, having swallowed two teaspoonfuls of the chloroform, at once, the previous night (Friday) at bedtime. In one of his letters, he told the party addressed to call, not later than 4 p. m. on Saturday, and see his corpse. If he took the chloroform at all, it is probable he did not do so until after eating the toast and tea at noon, when he had no difficulty in swallowing. Next day, Sunday, he was so well that medical attendance on him was discontinued. On Monday he sat up a while in his room.

During the night of August 18–19 he had diarrhœa, but the character of the stools is not known, as he was able to go to the water-closet. At 7 a. m. on August 19, he was found in his night dress, at the foot of the stairs, in a stupid, delirious condition, having slipped, probably, as he came out of the water-closet, which is at top of the stairs, and slid down to the bottom. He was taken back to bed. Then some blotches were noticed on the skin of his arms, but were attributed to injuries received in slipping down stairs. He continued delirious for a couple of hours.

At 11:30 a. m. I again saw him, and pronounced him dying. His appearance now was very remarkable. There was a well-marked icteroid hue of the skin of the face and neck. The jaundice was paler on the chest, and gradually faded away towards the extremities. Scattered all over the body, but more especially on the forearms and hands, were numerous purpuric spots varying in size from that of a large pin head up to one three inches long by two inches wide on the back of the right elbow joint. Neither the jaundice nor the blotches in the skin existed the previous day. Respiration was labored ; eyes closed ; conjunctivæ quite yellow ; pupils widely dilated ; no pulse at the wrists. Mouth partially open ; prolabia blue ; hands and feet cold. Temperature 36.70 in axilla. He was not entirely unconscious. He could and did swallow, and appeared to notice the pungency of brandy and ammonia. Once he moved his forearms inwards, and bent the fingers, claw-like, in a convulsive movement. This was the only convulsive action observed. The abdomen was flat. Pressure on the epigastrium and right hypochondrium caused him to moan aloud. Moving the body and lower extreminies also caused him to moan.

At 3 p. m. stertor, frequent turning of the head from side to side, and nystagmus set in. He died quietly, shortly after 4 p. m. During the two hours immediately before death, the icterus deepened.

As neither a chemical nor microscopical examination was made, I will not give any details of the autopsy, which did not throw any light on the cause of death. It is much to be regretted that the coroner did not order an analysis. Hæmorrhagic extravasations into the tissue of the heart, liver, stomach, etc., were observed.

I would beg to propound the following questions, and to solicit replies to them in this journal, particularly from those who devote special attention to chemistry and toxicology.

1. Would two teaspoonfuls, equal to at least three fluid drachms, of chloroform be likely to kill a healthy adult man, if taken at a dose, on an empty stomach?

2. Would a cup of tea and a small piece of toast eaten a little while before, prevent the fatal effect of a fluid ounce of chloroform swallowed soon after? Must not that quantity prove speedily fatal?

3. Even if the man did take, at a dose, three fluid drachms on Friday night, or a fluid ounce on Saturday afternoon, might the fatal effect be delayed until Tuesday forenoon, with entire absence of any evidence of chloroform in the system during Saturday, Sunday and Monday?

4. Can chloroform produce, in a man who had been drinking pretty freely of alcohol and absinthe for a couple of weeks before, icterus with hæmorrhage into the skin and internal organs?

I am aware that Dr. B. W. Richardson, of London, has seen "some of the characters of jaundice and some of the characters of scurvy" from the prolonged and frequent use of chloral in large doses, but he remarks: "The chloral, in undergoing decomposition within the body, divides into two products, the one chloroform, the other an alkaline formate, a soluble salt which makes the blood unduly fluid." Can chloroform alone do the same thing? Not that I am aware.

Phosphorus is the only agent known to me which can produce the appearances of the skin seen in this case shortly before death, unless chloroform can do so under the circumstances stated above. The writer is of opinion that phosphorus, in some form, was the cause of death. In the *British Medical Journal* for April 6, 1878, page 478, is given a case of phosphorus poisoning, in which jaundice was the only external evidence of the poison. On analysis, however, considerable phosphorus was found, in the form of phosphorous acid, which accounted for the absence of all luminosity. Under the microscope, characteristic appearances were found in the liver also. I believe if a chemical and microscopical examination were made in the present case, ample evidence of phosphorus must be found. There is a very close resemblance in the condition of the internal organs in the present case, with that seen in the case reported in the *British Medical Journal.*

The opinion of readers on this case would be very acceptable, I am sure, to Drs. M. O. Jones and N. Bridge, who had seen the case with me, as well as to Dr. D. R. Brower and to myself.

P. O'CONNELL, M.D.

CHICAGO, Sep. 4, 1879.

